# Supplementary material for: Homemade Kefir Consumption Improves Skin Condition—A Study Conducted in Healthy and Atopic Volunteers
Source: Foods. 2021 Nov 13;10(11):2794. doi: 10.3390/foods10112794 (PMC8622502; doi:10.3390/foods10112794)
Supplement: Supplementary file 1 [file foods-10-02794-s001.zip › foods-1449901-supplementary.pdf]

Supplementary Material

Supplemental Table S1 – Socio-demographic characteristics of study groups (relative frequency (%)).

**Sociodemographic characteristics**	**Healthy group (n = 33)**			**Atopic group (n = 19)**		
	**HK**	**H0**	***p* - value**	**AK**	**A0**	***p* - value**
Scholarity						
High School (12th grade), n (%)	0	0	0.225	4 (44.5)	0	0.167
Graduate, n (%)	12 (92.3)	15 (75.0)		3 (33.3)	5 (50.0)	
Master, n (%)	0	4 (20.0)		1 (11.1)	1 (10.0)	
Doctorate, n (%)	1 (7.7)	1 (5.0)		1 (11.1)	3 (30.0)	
Professional School, n (%)	0	0		0	1 (10.0)	
Career						
Employed, n (%)	1 (7.7)	2 (10.0)	0.822	4 (44.4)	5 (50.0)	0.809
University student, n (%)	12 (92.3)	18 (90.0)		5 (55.6)	5 (50.0)	
Residence area						
Urban, n (%)	8 (61.5)	18 (90.0)	0.051	6 (66.7)	9 (90.0)	0.213
Rural, n (%)	5 (38.5)	2 (10.0)		3 (33.3)	1 (10.0)	
Smoking habits						
Smoker, n (%)	4 (30.8)	2 (10.0)	0.121	1 (11.1)	1 (10.0)	0.622
Occasional smoker, n (%)	1 (7.7)	0		0	1 (10.0)	
Non smoker, n (%)	8 (61.5)	18 (90.0)		8 (88.9)	8 (80.0)	
Dairy consumption or substitutes						
Cow milk, n (%)	6 (46.2)	8 (40.0)	0.727	5 (55.6)	3 (30.0)	0.260
Natural yogurt, n (%)	11 (84.6)	17 (85.0)	0.976	6 (66.7)	8 (80.0)	0.510
Vegetable drink, n (%)	7 (53.8)	7 (35.0)	0.284	4 (44.4)	6 (60.0)	0.498
Alcohol consumption						
Never, n (%)	5 (38.5)	9 (45.0)	0.445	5 (66.7)	2 (20.0)	0.276
1 to 2 times/week, n (%)	7 (53.8)	11 (55.0)		3 (22.2)	6 (60.0)	
3 to 6 times/week, n (%)	1 (7.7)	0		1 (11.1)	2 (20.0)	

Groups were compared by Chi-Square test,with $p<0.05$ for statistical significance.

Supplemental Table S2 – Daily dietary intake characteristics of study groups (mean ± SD).

**Daily dietary intake characteristics**	**Healthy group (n = 33)**			**Atopic group (n = 19)**		
	**HK**	**H0**	***p* - value**	**AK**	**A0**	***p* - value**
Energy, kcal	1624 ± 469.1	1634 ± 592.5	0.941	1684 ± 315.8	1670 ± 344.0	0.870
Carbohydrates, %	43.7 ± 6.78	47.2 ± 5.97	0.224	50.4 ± 6.69	47.2 ± 5.07	0.191
Protein, %	23.7 ± 5.52	21.1 ± 4.87	0.197	22.5 ± 3.74	22.9 ± 3.98	0.806
Fat, %	32.3 ± 6.57	31.5 ± 5.24	0.912	27.1 ± 4.47	29.9 ± 4.45	0.165
Fiber, g	16.9 ± 4.78	16.4 ± 5.69	0.631	21.9 ± 4.80	18.8 ± 5.68	0.288

Water, L/day	2.18 ± 0.423	2.41 ± 0.459	0.071	2.39 ± 0.429	2.20 ± 0.286	0.347

SD – Standard deviation. HK – Healthy skin with kefir intake; H0 - Healthy skin without kefir intake; AK – Atopic skin with kefir intake; A0 – Atopic skin without kefir intake. Groups were compared using Mann-Whitney U-test, with $p < 0.05$ for statistical significance.

Supplemental Table S3 – Individual variation in skin parameters, between t0 and t8 (Wilcoxon standardized (Z) test statistic (*p*-value)).

**Skin parameters**	**Healthy group (n=33)**		**Atopic group (n = 19)**	
	**HK**	**H0**	**AK**	**A0**
TEWL (g/m²/h)				
Forearm	-2.412 (**0.016**)a	-0.597 (0.550)a	-2.666 (**0.008**)a	-1.274 (0.203)b
Leg	-1.014 (0.311)a	-0.784 (0.433)a	-2.666 (**0.008**)a	-2.701 (0.007)b
Forehead	-2.341 (**0.019**)a	-0.411 (0.681)a	-2.666 (**0.008**)a	-1.580 (0.114)b
Hydration (a.u.)				
Forearm	-0.039 (0.969)b	-2.380 (0.017)a	-2.675 (**0.007**)b	-1.429 (0.153)a
Leg	-0.774 (0.439)b	-1.069 (0.285)a	-2.668 (**0.008**)b	-0.358 (0.721)b
Forehead	-1.575 (0.115)b	-2.524 (0.012)a	-2.670 (**0.008**)b	-0.408 (0.683)b
Erythema (a*)				
Forearm	-2.271 (**0.023**)a	-2.782 (0.005)a	-2.310 (**0.021**)a	-0.561 (0.575)a
SCORAD Index	n.a.	n.a.	-2.666 (**0.008**)a	-1.682 (0.092)b

HK – Healthy skin with kefir intake; H0 - Healthy skin without kefir intake; AK – Atopic skin with kefir intake; A0 – Atopic skin without kefir intake. TEWL – Transepidermal Water Loss. Individuals were compared by Wilcoxon signed rank test, with $p<0.05$ for statistical significance. a - based on positive ranks (variable at t0 > variable at t8); b – based on negative ranks (variable at t0 < variable at t8).
